# Effects of parenting mode on student adaptability: the mediating effect of irrational beliefs

**DOI:** 10.1186/s12888-022-04222-5

**Published:** 2022-09-05

**Authors:** Kong Hua, Xu Hongwang, Deng Yujian, Wang Xuefeng, Zhang Wei

**Affiliations:** 1 Guangdong Ocean University, Zhanjiang, 524088 Guangdong Province People’s Republic of China; 2 Zhaotong University, Zhaotong, 657000 Yunnan Province People’s Republic of China

**Keywords:** Students’ adaptation, Emerging adulthood, Parenting mode, Mediating effect, Irrational beliefs

## Abstract

**Backgrounds:**

In the face of the new environment, different individuals have different reactions. Those who have good adaptability constantly establish individual self-efficacy through making friends and completing their studies, thus forming a good dependency with the university environment. However, individuals with poor ability to adapt to the new environment will have some bad phenomena, such as truancy, weariness and self denial. As a result, the students’ adaptations of to the growth environment where in universities are the important topics in recent years.

**Methods:**

Present study introduces irrational beliefs to investigate the effects of parenting mode on maladaptation of university students. The questionnaires based on simplified parenting mode (Chinese), irrational belief and adaptability were administered in a survey of 510 university students in Zhanjiang on October, 2021, the list of students of Guangdong Ocean University is taken as the sampling frame and determined by random sampling. Parenting mode was used as the independent variable, while the emotionally warm, overprotective and rejecting types were used as the indices. Further, the irrational beliefs including summary comments, awful beliefs and low tolerance to setbacks as well as maladaptation were included in the mediation model for analysis.

**Results:**

The results showed that the rejection parenting mode was negatively related with absolute requirements (*r* = − 0.143), and learning motivation (*r* = − 0.157), interpersonal adaptation (*r* = − 0.283) and physical and psychological adjustment (*r* = − 0.083). Overprotection was negatively correlated with absolute requirements (*r* = − 0.042) and interpersonal adaptation (*r* = − 0.042). The mediating effect of irrational beliefs (low tolerance to setbacks, awful beliefs and absolute requirements), the lower and upper limits of Bootstrap confidence interval were 0.135 and 0.461, respectively, excluding 0, which indicated that the mediating effect is true.

**Conclusion:**

Through the analysis of the data, this study believes that irrational beliefs such as low tolerance to setbacks, awful beliefs and absolute requirements mediate the effects on school adjustment. Negative parenting modes such as overprotection and rejection inculcate irrational beliefs, resulting in maladaptation of university students.

**Supplementary Information:**

The online version contains supplementary material available at 10.1186/s12888-022-04222-5.

## Background

Adaptation of students to the growth environment in universities and smooth transition to adulthood are important topics at the university level of development. Appropriate and effective adaptation of individuals to environment is called adaptive behavior [1]. Exposure to a new environment at the university leads to rapid acquisition of new learning habits and friendships, showing good adaptability of university students [2]. However, some students will develop maladaptation behaviors, including learning fatigue, skipping classes，depression and human communication disorders [3]. Therefore, adaptability of university students is a topic that has attracted increasing attention in recent years.

Some scholars believed that “adaptability of university students” refers to a psychological equilibrium that students develop after continuous behavioral adjustment to surrounding environment, classmates and teachers after admission into university [4]. It is also a process of physical and psychological adaptation to the local environment. Some scholars also pointed out that “adaptability of university students” requires changes in individual behavior to reach cognitive and emotional balance and comply with external environmental and regulatory standards [5]. It requires healthy adaptation to changing living and learning environment. Researchers reported that “adaptability of university students” is the process in which freshmen adjust psychologically and behaviorally to perform changing roles in the new university environment [6]. According to Watson [7], the adaptability of university students mainly refers to psychological and behavioral processes facilitating adaptation to the new environment by freshmen in the university based on personal adjustment and external support.

With respect to studies involving the role of factors associated with adaptability of university students, Bi et al. [8] investigated the effects of personality traits and interpersonal dynamics in a family on student adaptation. Zhu et al. [9] reported that students with different personality traits and family background adapt differently. Credé and Niehorster [10] summarized the factors influencing the adaptability of university students, including demographic characteristics, early achievements, experience in universities, self-assessment, personality traits, coping style, social support, and family relationship. Thus, Chinese and foreign studies investigating factors influencing the adaptability of university students mainly involve environmental and individual elements. Environmental factors mainly include family, interpersonal and school environment. Individual factors mainly refer to personality traits. External factors involve the quality of relationship with family members (eg, parenting mode) [11]. According to the Evolutionary Theory of Socialization, parents guide children to develop different behavioral modes of environmental adaptation based on parenting styles [12]. In other words, parental style have an important impact on the socialization of adult children. Parenting mode was firstly proposed by Diana Baumrind, a psychologist. From a psychological perspective, parenting mode refers to ordinary behavioral pattern of parents associated with raising and education of their children. It includes not only parenting behaviors, but also parenting emotions and attitudes [13]. Thus, parenting mode includes language and non-language behaviors or emotional atmosphere created by parents [14]. Arrindell et al. [15] divided parenting modes into warm emotional, overprotective and rejection types. The warm emotional type indicates prompt emotional recognition of children’s needs by parents by providing adequate love and supports, thereby ensuring two-way information flow, smooth communication and ordered and reasonable parent-child dependence [16–18]. Overprotection refers to parental recognition of children’s needs via excessive control and lack of space to exercise freedom of choice, thereby leading to one-way information flow and tense parent-child relationship. Rejection indicates parents’ insensitivity to the needs and appeals of their children, which hinders parent-child communication. Moreover, parents punish and excessively constrain children’s freedom. Researchers generally regard overprotection and rejection as negative parenting modes [19, 20].

Previous studies have shown that parenting styles and emotion regulation strategies can affect children intentionally or unintentionally [21]. Therefore, parenting style is conceptualized as a context that moderates the influence of specific parenting practices on the child [13, 22]. Compared with the other two parenting modes, the warm emotional type can induce development of rational beliefs and decrease maladaptation of university students. First, parents interact and communicate with children during parenting, resulting in shared positive emotional experiences. Therefore, parents can provide timely emotional feedback and material support to alleviate tension and anxiety in any maladaptation effectively [23]. Second, the warm emotional type contributes to a relatively close relationship between parents and children via two-way interaction and communication. As a result, children experience fewer negative emotions, such as insecurity and loneliness. Effective two-way interaction between parents and children in the new environment decreases emotional pressure associated with maladaptation [24, 25]. Third, experiencing parental emotions during stressful events is highly effective in enhancing psychological adaptation of children [22]. Increased parental encouragement and support can contribute to psychological health of children, including self-reliance [26]. Positive psychological traits and strategies to cope with crisis enable increased utilization of resources to resolve environmental stress, which, in return, can strengthen their self-efficacy. Positive effects of both authoritative parenting and relationship closeness on school performance were found for European Americans and, to some extent, second-generation Chinese [27, 28] In contrast, overprotected and rejected children experience negative emotions during growth and easily accumulate irrational beliefs. Irrational beliefs refer to absolute requirements and distorted perception of self, others, surrounding environment and objects [29].whereas indulgent and authoritative parenting shared greater parental warmth. In a university setting, which is beyond parental control, children encounter a new environment, make new friends and new interpersonal communication, and are forced to adapt to various tensions and anxieties caused by environmental changes [30]. To investigate the impact of previous beliefs on behavioral adaptation, this study introduced the concept of irrational beliefs to discuss the relationship between parenting mode and adaptability of university students outside parental control. The mechanism of parenting mode and its effect on the adaptability of university students and their development are discussed. Results provide a standard of reference for universities to implement accurate student management and prevent maladaptation.

What is the best parenting for healthy development and good school adjustment?According to previous studies, parenting based on demandingness, combined with responsiveness, is identify as the best parenting to promote healthy development, particularly in studies with middle-class European-American families [13, 31, 32]. Studies have also shown that parenting is closely related to cultural environment, for example，other studies identify the benefits of greater responsiveness, but without demandingness, mostly in studies in European countries as the beast parenting strategy for a healthy development [33, 34], including school achievement [35]. In general, a stable family structure, healthy development of family function and supportive parents are conducive to development of a positive personality in adolescence [36]. Further, children who have parents with higher education display better psychological health and less misconduct [37]. Based on the foregoing analysis, this study proposed the following hypothesis:

- H1. Negative parenting mode which includes rejection parenting mode and overprotection parenting mode can affect the student’s adaptation.
- H2. Negative parenting mode which include rejection and overprotection have a direct negative influence on the adaptability of university students.
- H3. Irrational beliefs have a complete mediating effect on students’ adaptation to negative parental rearing patterns.

### Research methods

#### Research subjects

A questionnaire survey was conducted in a university located in Zhanjiang on October, 2021 by using random sampling method. A total of 510 students participated in the survey and 510 completed questionnaires were received, showing a collection rate of 100%. The age range is 18–22 years old, mean is 18.37, standard deviation is 1.264. At the same time, since this survey does not consider the impact of ethnic attributes on students’ adaptation, the ethnic situation of the respondents is simplified into Han and ethnic minorities when collecting data. The specific sample composition is shown in Table 1.

**Table 1:** Descriptive analysis of demographic characteristics (*N* = 510)

Variables	Frequency	Percentage
Gender		
Male	232	45.5
Female	278	54.5
Nationality		
Han Nationality	496	97.3
Minority	14	2.7
Grade		
Freshmen	282	55.3
Sophomore	125	24.5
Junior	62	12.2
Senior	41	8.0
Politics Status		
League member	403	79
Ordinary citizen	88	17.3
CPC member (including probationary Party member)	19	3.7
Only child or not		
Yes	114	22.4
No	396	77.6
Stay-at-home children or not before university		
Half stay-at-home children	53	10.4
Complete stay-at-home children	22	4.3
No	435	85.3

### Research tools

#### Simplified parenting mode questionnaire for Chinese adolescents (S-EMBU-C)

In this study, the simple revised version [38] was administered. Since this study focuses on parenting mode, the parent questionnaire was integrated to generate 21 questions in three dimensions, including emotional warmth, overprotection and rejection. The questionnaire adopted the 4-level scoring method (1 = never and 4 = always). The higher total scores of factors indicate increasingly obvious parenting modes. In this study, the Cronbach’s α values associated with the three dimensions were 0.945, 0.823 and 0.915, respectively. The Cronbach’s α of the whole questionnaire was 0.848, the split half reliability of the whole questionnaire is 0.944，the internal consistency reliability of the questionnaire is high.

### Irrational belief survey (IBS) of university students

In this study, the irrational belief survey (IBS) compiled for university students was used [39]. IBS mainly includes 15 questions in four dimensions including summary comment, awful beliefs, absolute requirement and low tolerance to setbacks. The questionnaire used the 5-level scoring method. The higher score indicates stronger irrational beliefs (1 = strongly disagree and 5 = strongly agree). The Cronbach’s α values associated with the four dimensions of the questionnaire were 0.841, 0.706, 0.802 and 0.731, respectively. The Cronbach’s α of the whole questionnaire was 0.836, the split half reliability of the whole questionnaire is 0.854, the internal consistency reliability of the questionnaire is high.

### School adaptation questionnaire

The Student Adaptation to College Questionnaire (SACQ) compiled by Baker and Siryk in 1984 is used mostly for the evaluation of factors associated with adaptability of university students as well as scale development [40, 41]. This questionnaire comprises 67 questions in four dimensions: academic adjustment, social adjustment, personal/emotional adjustment and general institutional attachment. Combined with research practices, this study compiled a Chinese Student Adaptation to College Questionnaire based on SACQ, which contains 22 questions in five dimensions, including academic pressure, learning motivations, interpersonal adaption, school adjustment and physical-psychological adjustment. The questionnaire used a 5-level scoring method. The higher score indicates stronger adaptation (1 = strongly disagree and 5 = strongly agree). The Cronbach’s α values associated with the five dimensions were 0.841, 0.813, 0.789, 0.801 and 0.831, respectively. The Cronbach’s α of the whole questionnaire was 0.815, the split half reliability of the whole questionnaire is 0.851, the internal consistency reliability of the questionnaire is high. From Table 2, we can learn the total picture of major variables, this study contains three dimensions, each dimension contains several indicators.

**Table 2:** The dimension structure of questionnaires

Dimension	Index	Meaning
Parenting mode	Emotional warmth	Using seven questions to measure the behavior of parents in raising and educating their children，for example, when I succeed in what I do, my father/mother is very proud of me.
	Overprotection	Using eight questions to measure the behavior of parents in raising and educating their children, such as my parents asked me to explain to them what I did outside when I came home
	Rejection	Using six questions to measure the behavior of parents in raising and educating their children，for example, my parents often lose their temper with me without knowing why.
Irrational belief	Summary comment	Using five questions to measure the students’ unreasonable evaluation of themselves Such as If the teacher criticizes me, others will laugh at me/ Mother often gets angry with me when I don’t know why.
	Awful beliefs	Using three questions to measure the evaluation of negative events, such as bad things lead to long-term pain
	Absolute requirement	Using three questions to measure the belief that something will or will not happen, such as people should be respected by others
	Low tolerance to setbacks	Using four questions to measure the students’ attitudes in the face of setbacks, such as I can’t stand being rejected or rejected by my loved ones
School adaptation	Academic pressure	Using four questions to measure the emotional experience in academic such as I feel very difficult in my study
	learning motivations	Using four questions to measure the students’ attitudes and behaviors towards learning，such as I am not interested in studying when I entered college
	Interpersonal adaption	Using seven questions to measure the students’ cognition of interpersonal communication，such as I can get help from my college classmates when necessary
	School adjustment	Using four questions to measure the students’ adaptation, for example, I was often depressed when I entered college
	Physical-psychological adjustment.	Using three questions to measure the or example, I felt very happy when I entered college

### Data processing

Mediating effect refers to the impact of independent variable X on the dependent variable Y through the intermediate variable M [42]. Statistical analysis of major variables was performed using SPSS24.0 and Amos24.0 programs. According to the results of deviation from the common method, there are 8 factors with characteristic root higher than 1. The variance interpretation rate of the first common factor was 15.4%, which was smaller than the critical value (40%), which suggests that the deviation was not significant (Table 3).

**Table 3:** The explanation of total variation

Factor	Initial Eigenvalues			Extraction Sums of Squared Loadings			Rotation Sums Of Squared Loadings		
	Total	%of Variance	Cumulative %	Total	%of Variance	Cumulative %	Total	%of Variance	Cumulative %
1	7.481	24.937	24.937	7.481	24.937	24.937	4.632	15.439	15.439
2	3.356	11.186	36.123	3.356	11.186	36.123	3.664	12.214	27.652
3	2.219	7.395	43.519	2.219	7.395	43.519	2.945	9.818	37.47
4	1.838	6.128	49.646	1.838	6.128	49.646	2.83	9.434	46.904
5	1.444	4.812	54.458	1.444	4.812	54.458	1.817	6.055	52.959
6	1.071	3.57	58.028	1.071	3.57	58.028	1.294	4.314	57.273
7	1.012	3.373	61.401	1.012	3.373	61.401	1.238	4.128	61.401
8	1.006	3.152	64.153	1.006	3.152	64.153	1.211	4.018	64.323

The extraction method used is principal component analysis

## Results

### Analysis of parenting mode, irrational beliefs and school adjustment factors

#### Descriptive statistical analysis

The significance of differences between major demographic variables was analyzed. As shown in Table 4, we can learn that the perception of boys was weaker than that of girls regarding parenting mode. Girls were the most sensitive to emotional warm parenting mode.

**Table 4:** The descriptive analysis results of two independent groups (*N* = 510)

Gender (1 = male, 2 = female)		N	Mean	Std. Deviation	Std.Error Mean
WP	1	232	2.59	0.48	0.03
	2	278	2.69	0.50	0.03
R	1	232	2.04	0.34	0.02
	2	278	2.40	0.29	0.02
OP	1	232	2.10	0.33	0.02
	2	278	2.55	0.32	0.02

It can be seen from Table 5 that the emotional warmth type of education has a positive predictive effect on students’ adaptation. Refusal type and overprotective type had negative predictive effect on students’ adaptation (*p* <  0.05).

**Table 5:** Regression analysis of students’ adaptation among variables

Dependent variable	Independent variable	t	*p*
student’s adaptation	Emotional warm parenting mode	7.413	< 0.001
	Rejection parenting	−4.676	< 0.001
	Overprotection parenting;	−7.623	< 0.001

#### Validity analysis

The validity of this study mainly includes construct validity, convergent validity (Table 6) and the discriminant validity (Table 7).

**Table 6:** The convergent validity

Dimension	Item	STD	Unstd	S.E.	*t*-value.	P	SMC	C.R.	AVE
R	P16	0.793	1				0.63	0.80	0.57
	P13	0.807	1.054	0.065	16.167	***	0.65		
	P5	0.647	0.925	0.068	13.669	***	0.42		
OP	P19	0.631	1				0.40	0.67	0.40
	P9	0.617	0.941	0.1	9.385	***	0.38		
	P6	0.649	1.036	0.108	9.558	***	0.42		
WP	P14	0.839	1				0.70	0.84	0.63
	P12	0.79	0.956	0.053	17.883	***	0.62		
	P3	0.751	0.851	0.05	17.117	***	0.56		
Awful	I2	0.711	1				0.51	0.80	0.57
	I4	0.789	1.245	0.17	7.325	***	0.62		
	I6	0.754	1.191	0.162	7.338	***	0.57		
Absolute	I5	0.682	1				0.47	0.77	0.52
	I3	0.799	1.152	0.148	7.782	***	0.64		
	I1	0.683	0.989	0.131	7.556	***	0.46		
School Adaptation	B26	0.795	1				0.63	0.88	0.71
	B25	0.898	1.195	0.056	21.425	***	0.81		
	B24	0.836	1.081	0.053	20.338	***	0.70		
Interpersonal Adaptation	B11	0.858	1				0.74	0.89	0.73
	B12	0.888	0.939	0.039	23.815	***	0.79		
	B16	0.817	0.88	0.04	21.823	***	0.67		
Learning Motivation	B1	0.631	1				0.40	0.82	0.62
	B4	0.808	1.454	0.123	11.79	***	0.65		
	B3	0.905	1.639	0.14	11.729	***	0.82		
Learning Pressure	B9	0.828	1				0.63	0.88	0.71
	B6	0.628	0.743	0.056	13.225	***	0.81		
	B10	0.783	0.889	0.057	15.519	***	0.70		

*WP* emotionally warm parenting mode, *R* rejection parenting mode, *OP* overprotection parenting mode, *IB* irrational beliefs, *SA* student’s adaptation, *Std* Standardized estimates, *Unstd* Unstandardized estimates, *S.E.* the standard error of estimate, *SMC* Squared multiple correlation, *C.R.* composite reliability, *AVE* average of variance extracted, *t-value* critical ratio. See Supplementary file for the specific contents of the column “Item”

**Table 7:** The discriminant validity

	WP	OP	R	IB	SA
WP	0.79^**^				
OP	0.42^**^	0.63^**^			
RP	0.46^**^	0.46^**^	0.75^**^		
IB	0.23^**^	0.34^**^	0.34^**^	0.73^**^	
SA	0.37^**^	0.28^**^	0.38^**^	0.33^**^	0.82^**^
AVE	0.63	0.40	0.57	0.54	0.67

**means *p* < 0.01. *WP* emotionally warm parenting mode, *R* rejection parenting mode, *OP* overprotection parenting mode, *IB* irrational beliefs, *SA* student’s adaptation, *Ave* average of variance extracted. The diagonal line is the amount of variance variation extracted by Ave evaluation

By performing a confirmatory factor analysis using the Amos24.0 programs, we can find that the χ^2^/df = 2.142，less than 3，GFI = 0.901，greater than 0.9, AGFI = 0.917, greater than 0.9, RMSEA = 0.047,less than 0.08，NFI = 0.969, greater than 0.9, IFI = 0.926，greater than 0.9, CFI = 0.925, greater than 0.9. All values are within the acceptable standard range, indicating that the variables used in this study meet the requirements of the theoretical model.

Through CFA analysis of 9 potential variables, including warm parenting style, refusal parenting style, overprotective parenting style, extreme bad, absolute requirements, interpersonal adaptation, school adaptation, learning motivation and learning pressure，the load of all factors is between 0.6–0.9, and the average difference variation extraction is between 0.5–0.9 (Table 6). Meet the standards of Hair, et al. (2009) and Foretell and Larker (1981): 1. The factor load is greater than 0.5; 2. The extraction amount of mean variance variance is greater than 0.5 [43]. It can be seen that all variables in this study have good convergent validity.

Table 7 shows that there is a significant correlation between emotional warm parenting, irrational beliefs and students’ adaptation (*p* < 0.01). The absolute values of correlation coefficients are all less than 0.5, and are all less than the square root of the corresponding Ave. That is to say, there is a certain correlation between the potential variables, and there is a certain degree of discrimination between them, that is, the discrimination of the scale data is ideal.

### Mediating effect of irrational beliefs

**Fig. 1:**
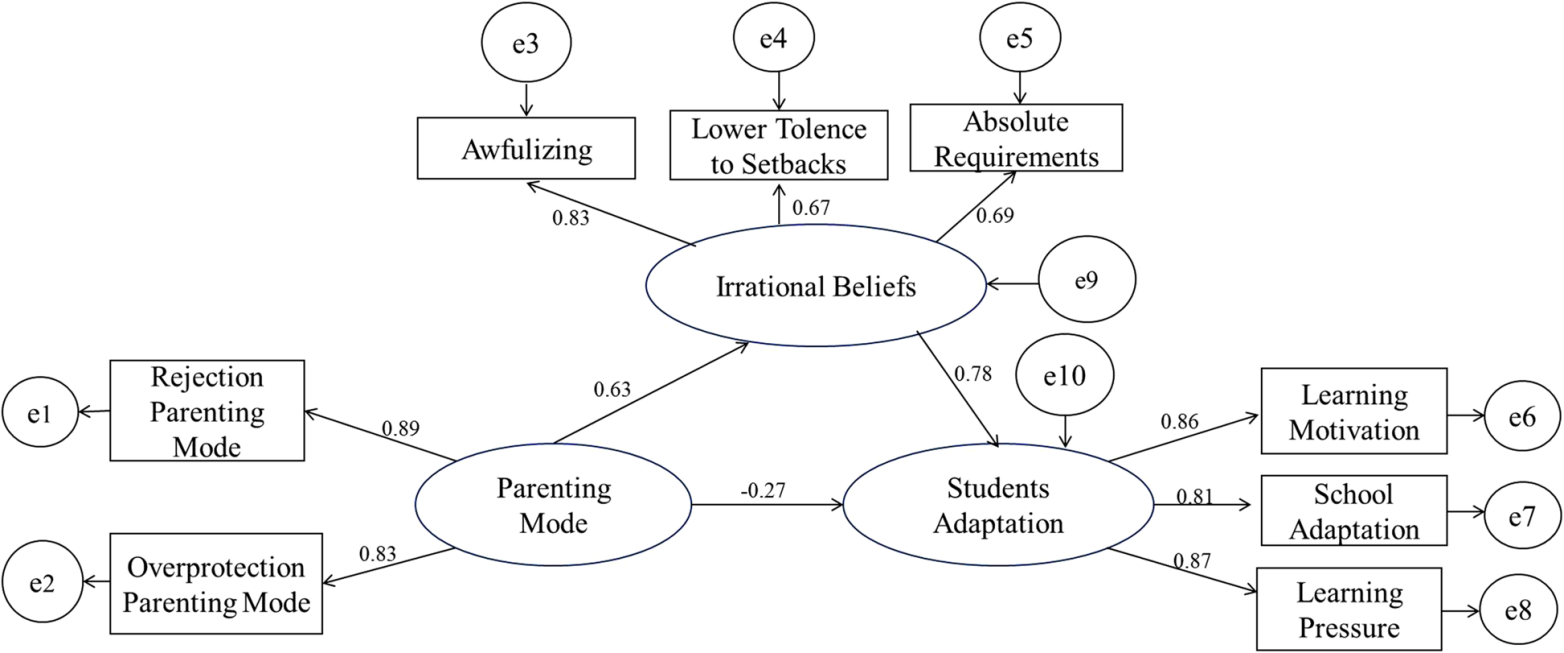
Mediating effect of irrational beliefs on the relationship between parenting mode and adaptability of university students

In this study, parenting mode was used as the independent variable, while emotionally warm, overprotective and rejecting types were used as indices. Further, irrational beliefs including summary comments, awful beliefs and low tolerance to setbacks as well as maladaptation were included in the mediation model for analysis. The simulation fitting result is relatively good. The standardization model of mediating effects based on pathway analysis is presented in Fig.1, and we can find the factor load.

A total of 5000 Bootstrap samples were analyzed using the Bootstrap method and the 95% confidence interval was selected to determine the general, direct and indirect mediating effects (Table 8). The total effect of negative parenting mode on students’ adaptation the lower and upper limits of the Bootstrap confidence interval were 0.253 and 0.396, excluding 0. Hence, the total effect is true. The lower and upper limits of Bootstrap confidence interval of direct effect were − 0.017 and 0.263, respectively, including 0. Hence, the direct effect is false. For the mediating effect of irrational beliefs (low tolerance to setbacks, awful beliefs and absolute requirements), the lower and upper limits of Bootstrap confidence interval were 0.135 and 0.461, respectively, excluding 0. Therefore, the mediating effect is true.

**Table 8:** The Bootstrap mediating effects of parenting on student’s adaptation

Effect	Standardization effect	95% CI	
		Lower limit	Upper limit
The total effect of negative parenting mode on students’ adaptation	0.036	0.253	0.396
The direct effect of negative parenting mode on students’ adaptation	0.092	−.0.017	0.263
The mediating effect of irrational beliefs on the relationship between negative parenting mode and adaptability of university students	0.261	0.135	0.461

## Discussion

From the above analysis，we can learn that the hypothesis we made have been verified。In other words，it suggests that parental warmth was negatively related to learning pressure, negative parenting modes including overprotection and rejection are associated with irrational beliefs awful beliefs, summary comments and absolute requirements are significantly positively correlated with school maladjustment. Parental warmth represents greater parental responsiveness and parental overprotection, greater demandingness. By contrast, parental rejection represents greater parental demandingness, but poor parental responsiveness [44, 45], which can cause students’ maladjustment [46]. The combination of parenting attitudes, emotional expression and parenting behaviors enhance parent-child interaction during development [47]. Thus, the parenting mode determines the conceptual and behavioral changes in children during growth and development of individual values. It is difficult to provide rational training if parents are overprotective, rejecting and crude, or criticize and control children unduly, and lack acceptance, understanding, forgiveness or respect for their children. Negative parenting modes can inculcate extreme and one-sided concepts in children, such as “any mistake will lead to serious consequences” and “I will be laughed at for mistakes”. These concepts will be solidified consciously and children find it difficult to accept individual failures and setbacks, resulting in irrational beliefs.

According to Bronfenbrenner, the microsystem, intermediate external system, and macrosystem represent a complete hierarchical system around individuals. Individual represents the core of the system and family is an important component of microsystem [48]. Parents are the first teachers of children and are important initial contacts. Family is the first site of individual socialization and represents the most direct and specific microenvironment for individual growth. It is an important element in individual adaptation [49]. As a structural system, family is an important place in childhood socialization. Each member is an important structural component and plays a unique role in the family [50]. Individuals learn social norms, cultural values and behavioral modes in families, while parents influence socialization of children via parenting mode [51]. Thus, the student behaviors reflect the parenting mode. Additional, warm parenting style has a positive impact on the whole life course of children [52]. University students are in early developmental stage of life, leaving families behind and facing a new living and learning environment. Arnett [53] also designated it as early adulthood characterized by individual instability, inadequate self-identity, self-concern, developmental transition and various possibilities. According to the eight-stage theory of personality development proposed by Erickson, university students are in the stage of transition from puberty to early adulthood. While insisting on self-identity, university students are eager to search for intimate relations with others and are extremely easy to exhibit maladaptation in interpersonal communication and psychological anxiety as well as discomfort [54]. Data suggest that parents and children are equally warm emotionally and children are provided with increasing number of opportunities for expression. Under such a parenting mode, children develop ability for empathy [55]. Following admission to the university, children from emotionally secure families develop good interpersonal relations. However, children experiencing overprotection and rejection often remain emotionally insecure and dependent, thus developing irrational beliefs such as absolute requirements. These children manifest maladaptation. University students in puberty are free from strict parental supervision and punishment, which decreases negative emotional experience. Besides, these students experience individual independence differently and the new connections with classmates and teachers increase their positive emotional experience. A decrease in negative emotional experiences with a concomitant increase in positive emotions facilitates adaptation of freshmen to campus environment, which is consistent with resource substitution theory and the law of conservation of energy [56]. Therefore, universities have an obligation to provide students with sufficient positive emotional experience upon admission, establish an all-inclusive teaching environment, increase their sense of acquisition and values, and improve self-reliance in individuals coping with difficulties and setbacks. The stronger the individual self-reliance, the more likely it is to develop acceptable behaviors for improved adaptability at the university level [57].

This study also found that irrational beliefs including poor tolerance to setbacks, awful beliefs and absolute requirements strongly mediate the relationship between negative parenting modes (overprotection and rejection) and maladaptation (insufficient learning motivation and poor interpersonal adaptation) of university students. This finding suggests that overprotection and rejection inculcates strong irrational beliefs. The study demonstrated that belief is an important factor influencing individual emotion and behavior. Many negative emotions and unhealthy psychological states are directly associated with irrational beliefs. As a result, students with profound and irrational beliefs easily experience adverse emotions compared with other students, resulting in academic difficulties and tense interpersonal relations. This mechanism of parenting mode contributes to maladaptation of university students observed in this study, suggesting that negative parenting mode does not directly influence the adaptability of university students, but contributes to their irrational beliefs. According to social cognitive theory of Ban Dula [58], self-cognition is influenced by individual attribution patterns, emotional and physiological responses, and the effects of others and verbal persuasion. Students’ irrational beliefs are mostly shaped by the past and emotional as well as psychological experiences. Children experiencing parental overprotection and rejection exhibit problems associated with interpersonal adaptation. University students who leave their parents behind have neither stable values nor well-developed self-cognition. This stage is an important element in strengthening students’ self-support system, shedding negative family influences gradually and establishing strong adaptability [59]. According to the social support theory, individuals can avoid negative effects of stress by receiving psychological and emotional support as needed [60]. Supportive behaviors of others not only help individuals physically and psychologically in daily life, but also lower subjective evaluation of individuals during severe crisis under stressful events. Therefore, schools can provide students with an established support system. For interpersonal adaptation, universities should focus on the management of different problems faced by different students and provide appropriate counseling and interventions. Universities should facilitate mutual assistance since communication and exchange among students are easier to create a strong atmosphere for interpersonal relations. To ensure psychological and self-adaptation, universities should provide a platform for diversified training and development, encourage students to set their own goals and independently chart out their careers. Universities should encourage different types of club activity, strengthen construction of a second classroom for students, provide multiple avenues for social interaction and arts and sports activities. Teachers can offer friendly support and assistance, ideological counseling and psychological guidance and thereby reinforce the university support system, strengthen the positive emotional experience and reduce the impact of irrational beliefs. Thus, the adaptability of university students can be improved [61].

This study suggests that irrational beliefs such as low tolerance to setbacks, awful beliefs and absolute requirements have a significantly positive correlation with maladaptation including insufficient learning motivation and poor interpersonal adaptation among university students. Personality is an inherent individual trait comprising a combination of stable and unique psychological qualities formed via interaction between genetics and environment. Individual hallmarks of personality include cognition, emotion and behavior [62]. The concept of individual cognition explains the formation and development of personality. Irrational beliefs can contribute to personality traits such as maladaptation among university students. Maladaptive behaviors can be generated by individual intolerance to setbacks or difficulties, or sheer pessimism [63]. Recent studies have shown that parental rearing patterns not only can significantly affect adolescents’ mental health [64], but also effect eating disorders and substance abuse [65]. Furthermore, students’ goal orientation, personality traits and parental rearing patterns are also closely related [66]. Although parental rearing patterns have various influences on young college students, universities can still do something for students. For example, universities should evaluate students’ parenting modes using surveys before beginning of term and then design different management strategies accordingly for accurate management, and improve management level and quality continuously. First, universities must accept students unconditionally, including their strengths and weaknesses. Second, universities should contribute to emotional experiences positively based on rational emotional therapy [67]. For example, universities can assist students with exploration of their potential positive emotions and strengths, promote development of positive qualities. They can facilitate exploration of personal values by establishing positive interpersonal relationships, setting up an accurate view of life, to inculcate the sense of success, formulate and realize goals based on their strengths. Thus, students can develop positive self-cognition.

### Strengths and limitations

This study provides a few standard recommendations to prevent and improve maladaptation of university students. In addition to developing positive parenting modes, parents need to establish robust relationship with children, create a warm, ordered, respectful and tolerant family atmosphere, and avoid crude one-way communication. Universities should strengthen connections with families, enhance parental counseling and communication skills. In particular, parents with poor relations with children are encouraged to participate in relevant professional counseling programs to provide change and support. Finally, schools should alter students’ irrational beliefs by strengthening the support system and rational emotional behavioral interventions to reduce maladaptation.

This study still has some limitations. First, it does not provide insights into causal relationships between variables since it uses cross-sectional data. Second, data were collected from self-reports of students. Further studies with improved experimental design are needed to increase the accuracy of results. Third, this study did not distinguish the parenting modes of father and mother and their effects on maladaptation, suggesting the need for further studies.

## Conclusions

A few major conclusions of the study are as follows:

1. Parenting mode can influence adaptability of university students.
2. Emotionally warm parenting mode strengthens the adaptability of university students. By contrast, negative parenting modes such as overprotection and rejection inculcate irrational beliefs, resulting in maladaptation of university students.

## Supplementary Information

**Additional file 1.**

## Data Availability

Data may be subject to third-party rights and restrictions. The data sets used and/or analyzed in the present study are available from the corresponding author on reasonable request.
