# Supplementary material for: Effects of parenting mode on student adaptability: the mediating effect of irrational beliefs
Source: BMC Psychiatry. 2022 Sep 5;22:592. doi: 10.1186/s12888-022-04222-5 (PMC9446861; doi:10.1186/s12888-022-04222-5)
Supplement: Supplementary file 1 — Additional file 1. [file 12888_2022_4222_MOESM1_ESM.docx]

**Supplementary file**

Questionnaire of Parenting Style on College Students

This project is about the investigation of the relationship between parenting style and college students 'adaptive behavior, mainly examining the impact of parenting style on college students' adaptive behavior. The main investigation content includes parenting style, college students 'irrational beliefs and college students' adaptive behavior. The answer has nothing to do with right or wrong, please answer truthfully. Regarding the parenting style section, where the father and the mother may be the same or different, please answer truthfully and separately. For the irrational belief and adaptive behavior part, please fill it out truthfully according to the actual situation. Thank you very much for your support.

Part 1: Basic information

Notes：This section is a topic choice, Please draw in parentheses before the corresponding answer according to your actual situation.

1.Your grade is：

( )Freshman

( )Sophomore

( )Junior

( )Senior

2.Your gender is:

( ) male

( )Female

3.Your political landscape is：

（）the masses

（）communist youth league member

（）CPC members (including probationary party members)

4.Your nation is

（）the Han nationality

（）the minority nationality

5.Are you the only child

( )yes

( )no

6.Staying behind before entering college

()Non-left-behind

()Half left behind

()Completely left behind

7.Your father's educational level is

( )Junior high school and below

( )High school / Technical secondary school / Junior college

( )Bachelor degree or above

( )No education experience

8.Your mother's educational level is

( )Junior high school and below

( )High school / Technical secondary school / Junior college

( )Bachelor degree or above

( )No education experience

Part 2

This part mainly investigates the parenting style of parents, please delimit in the corresponding form according to the actual situation

|  | | Never | Occasionally | Often | Always |
| --- | --- | --- | --- | --- | --- |
| P1 | My parents were proud of me when I did successful. |  |  |  |  |
| P2 | My parents often gets angry at me without me knowing the reason |  |  |  |  |
| P3 | Father praised me. |  |  |  |  |
| P4 | I hope my parents does not worry too much about what I am doing. |  |  |  |  |
| P5 | My parents tend to punish me more than I deserve |  |  |  |  |
| P6 | My parents asked me that I had to go home to show them what I had done outside. |  |  |  |  |
| P7 | I think my parents try to make my teenage life more meaningful and colorful. |  |  |  |  |
| P8 | My parents often criticize me in front of others for being lazy and useless. |  |  |  |  |
| P9 | My parents didn't allow me to do things that other children could do because she was afraid I would happen. |  |  |  |  |
| P10 | My parents always tries to encourage me and make me the best. |  |  |  |  |
| P11 | I think my parents’ concern about my possible accident is exaggerated and excessive. |  |  |  |  |
| P12 | When something goes wrong, I can feel my parents try to encourage me and comfort me. |  |  |  |  |
| P13 | I'm often used as a scapegoat or black sheep at home. |  |  |  |  |
| P14 | I can feel that they love me through my parents’ words and expressions. |  |  |  |  |
| P15 | My parentsr often allows me to go where I like it, and she doesn't worry too much. |  |  |  |  |
| P16 | My parents often treat me in an embarrassing way. |  |  |  |  |
| P17 | My parents often allows me to go where I like it, and she doesn't worry too much. |  |  |  |  |
| P18 | I felt that there was a warm, thoughtful and affectionate feeling with my parents. |  |  |  |  |
| P19 | My parents have strict restrictions on what I should do and never give in. |  |  |  |  |
| P20 | Even for my minor mistakes, My parents also punished me. |  |  |  |  |
| P21 | My parents always affects what I should wear or what to dress like. |  |  |  |  |

Part 3

This part mainly investigates irrational beliefs, please delimit in the corresponding form according to the actual situation

|  | | Very Opposition | Opposition | Neutral | Strongly Agree | Agree |
| --- | --- | --- | --- | --- | --- | --- |
| I1 | Bad people should be blamed or punished accordingly |  |  |  |  |  |
| I2 | Unsatisfactory things will inevitably lead to lasting pain |  |  |  |  |  |
| I3 | People should be respected by others |  |  |  |  |  |
| I4 | One must be very capable and successful in all ways, or it will be too worthless |  |  |  |  |  |
| I5 | In any case, others should treat me fairly |  |  |  |  |  |
| I6 | Any mistake can lead to a great disaster |  |  |  |  |  |
| I7 | I can't stand making a fool of myself. It loses face |  |  |  |  |  |
| I8 | Sometimes I feel like the most unfortunate person in the world |  |  |  |  |  |
| I9 | I can't stand being rejected or rejected by someone I love |  |  |  |  |  |
| I10 | If the current situation is not ideal, the future will not be good |  |  |  |  |  |
| I11 | I couldn't bear to put myself in a stressful and stressful situation |  |  |  |  |  |
| I12 | He who made great mistakes had no hope |  |  |  |  |  |
| I13 | I can't tolerate bad performance or failure on important tasks |  |  |  |  |  |
| I14 | If the goals are not all achieved, then the efforts will be all in vain |  |  |  |  |  |
| I15 | If the teacher criticizes me, others will laugh at me, too |  |  |  |  |  |

Part 4

This section mainly investigates school adaptive behavior,please delimit in the corresponding form according to the actual situation.

|  | | Very Opposition | Opposition | Neutral | Strongly Agree | Agree |
| --- | --- | --- | --- | --- | --- | --- |
| B1 | After entering the university, I think the study is very important |  |  |  |  |  |
| B2 | After entering the university, I was not interested in studying |  |  |  |  |  |
| B3 | After entering the university, I was very enthusiastic about my study |  |  |  |  |  |
| B4 | After entering the University, I took my study very seriously |  |  |  |  |  |
| B5 | After entering college, it was very painful for me to study |  |  |  |  |  |
| B6 | I find it very difficult to study in University |  |  |  |  |  |
| B7 | I think I may have problems with my learning method |  |  |  |  |  |
| B8 | In my study, I feel a little overwhelmed |  |  |  |  |  |
| B9 | I felt at a loss what to do to study in college |  |  |  |  |  |
| B10 | The pressure of studying in university is too great |  |  |  |  |  |
| B11 | I have a good relationship with my classmates |  |  |  |  |  |
| B12 | I have a lot of friends in college |  |  |  |  |  |
| B13 | In college, I feel like I am very popular |  |  |  |  |  |
| B14 | In college, I feel like I am very popular |  |  |  |  |  |
| B15 | In college, I often play with my classmates |  |  |  |  |  |
| B16 | In college, I was generally estranged from other people |  |  |  |  |  |
| B17 | In college, I have more intimate friends |  |  |  |  |  |
| B18 | I am satisfied with my personal relationships in my university |  |  |  |  |  |
| B19 | When I need it, I can get help from my college classmates |  |  |  |  |  |
| B20 | After entering college, I often sleep badly |  |  |  |  |  |
| B21 | After entering college, I was often energetic |  |  |  |  |  |
| B22 | After entering the university, I was very happy |  |  |  |  |  |
| B23 | After entering college, I often had dizziness or a headache |  |  |  |  |  |
| B24 | After I enter college, I often feel nervous |  |  |  |  |  |
| B25 | After entering college, I was often depressed |  |  |  |  |  |
| B26 | After entering college, I often lose my temper easily |  |  |  |  |  |
